# Unpublished Contributions

**Published:** 1841-09

**Authors:** 


UNPUBLISHED CONTRIBUTIONS.

*Meteorological Observations.* Dr. C. B. Guthrie of Logan, Ohio, has favored us with the monthly results of one year's observations on the thermometer, which we were about to publish, when by taking their average, we discovered that they gave a mean annual temperature of only 47° the lat. being 39° 30'. This being at least 6° below what that latitude should give, we at once perceived something wrong. This was nothing else than the making of the observations, at such hours as not to give the highest temperature of the day. It is very desirable that all observations should be made, by different observers, at the same times, to-wit, early in the morning, and between 2 and 3 o'clock in the afternoon, otherwise their tables are not comparable.

*Wound of the head and escape of a portion of brain—recovery.* The same gentleman has given us the history of a case, in which, from the kick of a horse against the head of a boy four years old, a portion of the frontal bone, was driven in—the scalp and dura mater lacerated—and a considerable quantity of brain suffered to escape. The fragment of bone was raised and left *in situ.* In a week his little patient was running about the house. Such a recovery does credit to the surgeon in attendance; but the case does not present enough of novelty to call for its publication.

*Tracheotomy—extraction of a grain of corn,* 21 *days after its introduction into the windpipe.* Dr. M. G. Kreider has favored us with the history of such a case. The symptoms were those of laryngitis, and he had no suspicion of a foreign body in the windpipe till three weeks after the accident. He immediately operated, and his little patient was restored. A more extended notice of the case seems unnecessary.

*Abdominal Tumour.* Dr. Harlon Hard of Waldo, Ohio, has narrated to us the symptoms of a remarkable tumour of this kind, the nature of which is doubtful. Whenever the case may terminate and its history be completed, it will be worthy of publication.

*Tic Douloureux.* The same gentleman has given us the history of a case of this hopeless malady, in which four operations had been performed on branches of the trifacial nerve, with no more than temporary relief. All the usual medicines had been prescribed in large doses, without effect. We should be happy to publish an account of the cure of his patient.

*Imperfect palsy of the limbs of children.* From Dr. Robert S. Newton of Gallipolis, Ohio, we have received the notes of a case of this kind, in which the paralysis occurred in the progress of a fever. His patient recovered. He mentions that several other cases of the same kind presented themselves about the same time. We hope to get a detailed account of the whole, and therefore do not publish that which has been sent us. Our readers will be reminded by these cases, of those published in the 2d vol. of the Journal as occurring in Xenia, Ohio.

*Colchicum in Tetanus.* Dr. Davius Maxon of Gallipolis, has given us a short notice of a case which he regarded as tetanus, not produced by local injury, in which two dram doses of the wine of colchicum appeared to be the chief cause of the recovery of his patient. As the symptoms were not altogether diagnostic of that disease, we consider this notice sufficient.

*Fevers of Illinois.* We thank Dr. Jethro Hatch of Vermillionville, Illinois, for a letter containing some interesting information relative to the autumnal fevers of that region—not, however, drawn up with the systematic care and fulness necessary for publication *in extenso*, nor susceptible of abridgement.

*Sympathetic effects of Uterine diseases.* Dr. Allen Kimbal of Fort Henderson, Alabama, has favored us with a brief report of eight cases intended "to show the influence of uterine diseases over the general health of females in Southern climates." These reports have not the fulness that is desirable; in some of them the uterine affection seems to have been *secondary;* and, in general character, the whole of them are identical or exceedingly similar to cases which constantly occur further North. While, therefore, we thank our correspondent for his extended communication, and hope to hear from him again, we feel restrained from its publication. D.

